# Prophylactic Olive Leaf Tea as a Nutraceutical Strategy: Tumor Suppression and Systemic Protection

**DOI:** 10.3390/cimb47110926

**Published:** 2025-11-06

**Authors:** Hatice Gumushan Aktas, Awat Omar Sabr, Cigdem Gungormez, Mirac Uckun, Hidir Sulak, Ahmet Ozkaya, Jihad Haji Saleh, Ertan Yologlu, Belkis Tekguler, Ulas Alabalik

**Affiliations:** 1 Biology Department, Faculty of Arts and Science, Harran University, Sanliurfa 63050, Türkiye; 2 Medical Microbiology Department, Faculty of Science and Health, Koya University, Erbil 44001, Iraq; awat.omar@koyauniversity.org; 3 Medical Biology Department, Faculty of Medicine, Siirt University, Siirt 56100, Türkiye; gungormezcigdem@gmail.com; 4 Department of Food Engineering, Faculty of Engineering, Adıyaman University, Adıyaman 02040, Türkiye; miracuckun@gmail.com; 5 Sanliurfa Youth and Sports Provincial Directorate, Sanliurfa 63210, Türkiye; dytsulak@gmail.com; 6 Department of Chemistry and Chemical Processing Techniques, Vocational School of Technical Sciences, Adıyaman University, Adıyaman 02040, Türkiye; aozkaya01@gmail.com; 7 Nursing Department, Safeen Technical and Vocational Institute, Erbil 44001, Iraq; jihad.7ajy@gmail.com; 8 Department of Mathematics and Science Education, Faculty of Education, Adıyaman University, Adıyaman 02040, Türkiye; eyologlu@adiyaman.edu.tr; 9 Department of Food Engineering, Faculty of Engineering, Ondokuz Mayis University, Samsun 55270, Türkiye; belkisg@omu.edu.tr; 10 Pathology Department, Faculty of Medicine, Dicle University, Diyarbakır 21280, Türkiye; ulasalabalik@gmail.com

**Keywords:** olive leaf tea, breast cancer, hepatoprotective effect, Ehrlich Ascites Tumor, microRNAs

## Abstract

Olive leaf tea (OLT), rich in phenolics, exhibits antioxidant, anti-inflammatory, and potential anticancer effects; however, the in vivo efficacy remains unclear. This study evaluated the chemopreventive and systemic effects of OLT in a murine Ehrlich Ascites Tumor (EAT) model, with a focus on the treatment timing. OLT was prepared by aqueous infusion and characterized for total phenolic content (TPC: 25.74 mg GAE/g), DPPH scavenging (197.88 µmol TE/g), FRAP activity (81.23 µmol Fe^2+^/g), and LC-MS/MS profile (oleuropein 77.6%). Mice received OLT orally before or after tumor inoculation. Prophylactic OLT reduced EAT cell counts (from 31.48 × 10^7^ to 21.15 × 10^7^), ascites volume (from 4.58 to 2.98 mL), elevated miR-155-5p (14.34-fold), normalized ALT/AST, and restored hepatic antioxidants without histopathological damage. Co-treatment with 5-FU preserved efficacy while reducing hepatotoxicity. In conclusion, OLT provides timing-dependent anticancer and systemic protective effects in the EAT model, supporting its potential as a cost-effective nutraceutical for cancer prevention and adjunctive therapy.

## 1. Introduction

Worldwide, cancer, a significant global health burden, is responsible for approximately 9.7 million deaths in 2022, while breast cancer is still the most frequently diagnosed malignant tumor among women [1]. Despite the advancements in diagnostics and therapeutics, preventive strategies have been receiving prominence due to their accessibility and potential for diminishing cancer risk [2].

Oxidative stress is caused by excessive accumulation of reactive oxygen species (ROS). It plays an important role in the pathogenesis of various chronic conditions, including cardiovascular diseases, diabetes, and cancer, by damaging cellular components such as DNA, proteins, and lipids [3]. The body protects itself from reactive oxygen species (ROS) through innate antioxidant enzymes, including superoxide dismutase, glutathione peroxidase, and catalase. In addition to these endogenous systems, dietary polyphenols significantly contribute to the neutralization of reactive oxygen species (ROS). Olive leaf tea, a significant source of phenolics such as oleuropein and hydroxytyrosol, exhibits robust antioxidant activity and protective advantages in many disease models linked to oxidative stress [4].

The olive tree (*Olea europaea* L.), particularly its leaves, has been used in medicine for many years across the Mediterranean region. Olive leaf extracts are officially recognized by the European Medicines Agency as traditional herbal medicinal products for their therapeutic properties [5]. Extensive research has demonstrated a broad spectrum of biological activities associated with olive leaf extracts, including antihypertensive, antidiabetic, antimicrobial, antiviral, anti-inflammatory, and anticancer effects [6,7,8,9,10]. These effects are attributed mainly to the presence of bioactive polyphenols such as oleuropein, hydroxytyrosol, and verbascoside [11].

Oleuropein, the primary secoiridoid found in olive leaves, has been widely studied for its anticancer potential. It has been pointed out to inhibit cell proliferation, induce apoptosis, and block invasion in various cancer cell lines, including breast, prostate, and leukemia [12,13,14]. In breast cancer cells such as MCF-7 and MDA-MB-231, it has been found that oleuropein triggered apoptosis through mitochondrial pathways and suppressed NF-κB signaling [13]. In SH-SY5Y neuroblastoma cells, it induced cell cycle arrest and modulated key apoptotic gene expression [15]. It is noteworthy that oleuropein exhibited selective cytotoxicity, maintaining antioxidant behavior in normal cells and acting as a prooxidant in cancer cells [16].

Besides oleuropein, olive leaves are rich in many bioactive molecules, including phenolic acids, luteolin derivatives, rutin, hydroxytyrosol, tyrosol, diosmin, and verbascoside [17,18,19]. These bioactive molecules have been linked to positive impacts on conditions such as neurodegenerative disorders (including Alzheimer’s and Parkinson’s diseases), metabolic syndrome, diabetes, and obesity [20,21]. Furthermore, olive leaf extract has demonstrated efficacy in reducing blood pressure, promoting coronary artery dilation, and exerting anticancer activity against leukemia, colon cancer, erythroleukemia, and renal adenocarcinoma [22,23,24].

Recent in vivo investigations have begun to substantiate the anticancer potential of olive leaf and phenolic compounds. For example, in silico supported reviews by Gervasi et al. (2024) emphasized the efficacy of oleuropein and hydroxytyrosol in suppressing tumor growth in vivo, while showing the dual role of these phenolics as both antioxidants and prooxidants depending on content and concentration [25]. A broader meta-analysis has confirmed the antitumor activity of olive leaf extracts, especially oleuropein and oleocanthal, with demonstrated efficacy in breast and colon cancer xenograft models, and remarked synergy with conventional chemotherapeutics in reducing tumor burden in vivo [26].

MicroRNAs (miRNAs) are small, non-coding RNAs, ~22 nucleotides in length, that regulate gene expression at the post-transcriptional level, affecting 30–60% of the human genome. They are actively involved in essential cellular mechanisms such as differentiation, proliferation, programmed cell death, epithelial-mesenchymal transition (EMT), formation of new blood vessels, and migration [27]. Aberrant miRNA expression profiles have been strongly linked to tumorigenesis, functioning either as oncogenes or tumor suppressors [28]. miR-155 is a good example of this paradoxical role. While it exhibits an oncogenic activity in cancers such as leukemia and lymphoma, many miRNAs, including miR-155, are downregulated in cancers such as breast, lung, ovarian, and colorectal cancers [29], making them promising biomarkers for cancer diagnosis, prognosis, and treatment response assessment.

Various studies suggest that microRNAs, including miR-155, play important roles also in liver diseases [30,31]. miR-155 expression was elevated in alcoholic liver disease, and its knockout protected mice from alcohol-induced damage [32]. In contrast, miR-155-deficient mice showed increased ALT levels on a high-fat diet, indicating its role in liver homeostasis and lipid metabolism [33]. In drug-induced liver injury, no change in miR-155 expression was observed [34]. In patients with hepatitis B, blood miR-155 levels were lower than in healthy individuals, but higher in those with elevated ALT. This was likely reflecting immune activation rather than a direct correlation [35]. Another study demonstrated a positive correlation between miR-155 and ALT in patients with chronic hepatitis B. It has been reported that chronic HBV may inhibit miR-155 expression, resulting in the dysregulation of miR-155 in NK cells [36]. These conflicting findings indicate the need for further research.

Despite substantial evidence supporting the antioxidant and anticancer potential of olive leaf extracts and their major phenolics, such as oleuropein and hydroxytyrosol, most existing reports are limited to in vitro or in silico observations, with scarce in vivo data addressing the timing and systemic safety of such formulations. Furthermore, no previous study has investigated the potential link between OLT administration and modulation of hepatic microRNA expression, particularly miR-155-5p, which plays a dual role in tumor suppression and immune regulation. Therefore, this study fills an important gap by providing the first in vivo evaluation of the timing-dependent chemopreventive efficacy and systemic protective profile of olive leaf tea (OLT) in an Ehrlich Ascites Tumor model. Through combined biochemical (ALT, AST, CaE, GST, GSH), molecular (miRNA expression), and histopathological analyses, our findings offer new insights into how a simple dietary formulation can modulate oxidative stress, hepatic microRNA expression, and multi-organ health, supporting the translational potential of OLT as a prophylactic nutraceutical against cancer development.

## 2. Materials and Methods

### 2.1. Preparation of Olive Leaf Tea

The olive leaves used in this study were collected from mature olive trees located in a privately owned family orchard in Mersin, Türkiye (36°45′13.8398″ N, 34°23′7.9814″ E). The orchard is maintained under conventional cultivation practices without the use of pesticides. The collected plant material was taxonomically authenticated by Prof. Dr. Erkan Yalçın at Ondokuz Mayıs University (Herbarium No: OMUB-8817). After washing with distilled water, the olive leaves were air-dried at room temperature for 48 h, oven-dried at 40 °C with continuous airflow (≈1 m/s) for 24 h, and stored in airtight amber glass containers at 22 ± 2 °C in the dark to preserve phenolic stability until daily use. The dried olive leaves were manually crushed into small fragments (≈2–3 mm) using a sterile porcelain mortar and pestle to avoid heat generation and preserve phenolic integrity before infusion. For OLT preparation, 5 g of dried leaves were crushed and infused in 100 mL of distilled water (95 °C) for 10 min (50 mg/mL) in a covered glass beaker, which remained closed during steeping and cooling to minimize the loss of volatile metabolites before filtration. After cooling, the infusion was sequentially filtered through a 0.45 μm syringe filter to remove particulates and then through a 0.2 μm sterile syringe filter to ensure sterility. The final filtrate obtained after sequential filtration was defined as OLT and used for all subsequent analyses.

### 2.2. Evaluation of Antioxidant Properties and Phenolic Profile of Olive Leaf Tea

#### 2.2.1. Chemicals

All reagents used were of analytical grade unless otherwise stated. Folin–Ciocalteu reagent (≥98%) was obtained from Carlo Erba Reagents SAS (Val-de-Reuil, Normandy, France). 2,2-Diphenyl-1-picrylhydrazyl (DPPH, ≥97%), 2,4,6-tripyridyl-s-triazine (TPTZ, ≥99%), ferric chloride hexahydrate (FeCl_3_·6H_2_O, ≥98%), ferrous sulfate heptahydrate (FeSO_4_·7H_2_O, ≥99%), sodium carbonate (Na_2_CO_3_, ≥99.5%), glacial acetic acid (≥99.7%), acetate buffer components (sodium acetate ≥99%), hydrochloric acid (HCl, 37%), gallic acid monohydrate (≥98%), and 6-hydroxy-2,5,7,8-tetramethylchroman-2-carboxylic acid (Trolox, ≥97%), formic acid (≥98%), and methanol (≥99.9%, GC) were purchased from Sigma-Aldrich (St. Louis, MO, USA).

All phenolic standards used for LC–MS/MS analysis (vanillic acid, syringic acid, fumaric acid, caffeic acid, hydroxybenzoic acid, salicylic acid, oleuropein, quercetin, luteolin, acetohydroxamic acid, catechin hydrate, myricetin, kaempferol, resveratrol, hydroxycinnamic acid, protocatechuic acid, phloridzin dihydrate, cumin, thymoquinone, ellagic acid, butein, naringenin, silymarin, and alizarin) were purchased from Sigma-Aldrich (St. Louis, MO, USA; ≥95–99% purity, HPLC-grade).

Ultrapure water was used in all experiments and solution preparations.

#### 2.2.2. LC-MS/MS Analysis of Phenolic Compounds

Phenolic compound quantification was performed at the Central Research Laboratory of Harran University (HUBTAM) using a UHPLC system coupled with tandem mass spectrometry (LC-MS/MS; Shimadzu, Kyoto, Japan) equipped with an Inertsil ODS-4 C18 column (2.1 × 50 mm, 2 µm particle size, GL Sciences Inc., Tokyo, Japan) and a binary gradient system (Pumps A and B, LC-20ADXR). The mobile phase A consisted of 0.1% (*v*/*v*) formic acid in ultrapure water, and mobile phase B consisted of 0.1% (*v*/*v*) formic acid in methanol. The total flow rate was set to 0.40 mL/min with the following gradient program: 5% B at 0 min, increased to 95% B at 4 min, held for 3 min, returned to initial conditions at 7.01 min, and equilibrated for 5 min before the next injection. The column temperature was maintained at 40 °C using a CTO-10ASvp oven (Shimadzu Corporation, Kyoto, Japan), and the injection volume was 2 µL. The LC effluent was introduced into the mass spectrometer via an electrospray ionization (ESI) source, operated in either positive or negative ionization mode, depending on the compound. The *m*/*z* range was scanned from 100 to 1000, with a nebulizing gas (N_2_) flow of 3 L min^−1^, desolvation gas flow of 15 L min^−1^. Interface temperature, DL temperature, and Heat block temperature were 350 °C, 250 °C, and 400 °C, respectively.

The following phenolic compounds were analyzed and quantified based on their retention times, with peak areas normalized to authentic standards: vanillic acid, syringic acid, fumaric acid, caffeic acid, hydroxybenzoic acid, salicylic acid, oleuropein, quercetin, luteolin, acetohydroxamic acid, catechin hydrate, myricetin, kaempferol, resveratrol, gallic acid, hydroxycinnamic acid, protocatechuic acid, phloridzin dihydrate, cumin, thymoquinone, ellagic acid, butein, naringenin, silymarin, alizarin. Calibration curves were generated for each compound using external standards, and the results were expressed as mg/kg of olive leaves and as a percentage of the total identified phenolic amount.

#### 2.2.3. Determination of Total Phenolic Content (TPC)

Total phenolic content was determined using the Folin–Ciocalteu method as described before [37], with minor modifications. Based on our preliminary trials, the procedure was optimized by adjusting the sample-to-reagent ratio and incubation conditions to achieve maximum absorbance and reproducibility. Briefly, 0.05 mL of the OLT was mixed with 0.05 mL of Folin–Ciocalteu reagent (1:1, *v*/*v*). After 5 min, 0.25 mL of freshly prepared 10% (*w*/*v*) Na_2_CO_3_ solution in ultrapure water was added. The mixture was incubated for 2 h at room temperature in the dark, and the absorbance was measured at 760 nm using a spectrophotometric microplate reader (MultiSkan IT, Thermo Fisher Scientific, Waltham, MA, USA). A reagent blank containing all components except the sample extract was used as the reference. All analyses were performed in triplicate, and the mean ± standard deviation values were reported. The total phenolic content was expressed as mg gallic acid equivalent (GAE) per gram of dried plant material (mg GAE/g dry sample) based on a gallic acid calibration curve (concentration range: 20–100 µg/mL, y = 0.0105x + 0.0106, R^2^ = 0.9995).

#### 2.2.4. DPPH Radical Scavenging Activity

The antioxidant activity of the OLT was determined using the DPPH radical scavenging assay, originally developed by Blois (1958) [38] and adapted for microscale conditions according to Brand-Williams et al. (1995) [39]. In this microscale protocol, 0.05 mL of OLT extract was mixed with 0.1 mL of 0.1 mM DPPH solution in methanol (1:2, *v*/*v*). The reaction mixture was incubated for 2 h at room temperature in the dark, based on preliminary optimization studies that identified this duration as sufficient for reaction equilibrium and maximum absorbance stability. The absorbance was measured at 515 nm using a microplate spectrophotometer (MultiSkan IT instrument, Thermo Fisher Scientific, Waltham, MA, USA). A methanol-only control containing the DPPH solution without the sample extract was used to determine the baseline absorbance. All analyses were performed in triplicate, and results were expressed as µmol Trolox equivalents per gram of dried plant material (µmol TE/g dry sample) based on a Trolox standard calibration curve (concentration range: 0.05–0.9 mM, y = 80.024x + 1.8358, R^2^ = 0.9943).

#### 2.2.5. Ferric Reducing Antioxidant Power (FRAP) Assay

FRAP activity was determined according to the method described by Benzie & Strain (1996) with slight modifications [40]. The FRAP reagent was freshly prepared by mixing 300 mM acetate buffer (pH 3.6), 10 mM TPTZ in 40 mM HCl, and 20 mM FeCl_3_·6H_2_O in a ratio of 10:1:1 (*v*/*v*/*v*). A 0.95 mL aliquot of the OLT sample was mixed with 0.05 mL of the FRAP reagent and incubated at 37 °C for 5 min. The absorbance was measured at 593 nm using a microplate spectrophotometer (MultiSkan IT instrument, Thermo Fisher Scientific, Waltham, MA, USA). A reagent blank containing only the FRAP reagent (without the OLT sample) was used to determine the baseline absorbance. All analyses were carried out in triplicate. The antioxidant capacity was calculated from a ferrous sulfate (FeSO_4_·7H_2_O) calibration curve (concentration range: 0.05–0.9 mM, y = 2.7502x + 0.0624, R^2^ = 0.9983), and expressed as µmol Fe^2+^ equivalents per gram of dried sample.

### 2.3. Animals

In the experiments, 54 male Balb/c mice, 8 weeks old and averaging 25 g in weight, were used. The animals were housed in standard laboratory conditions (4 mice per polycarbonate cage of 0.019 m^3^) with a 12 h light/dark cycle and an ambient temperature of 22 ± 4 °C. Throughout the experiment, all mice had ad libitum access to tap water and standard laboratory feed (DSA Agrifood Products, Kirikkale, Türkiye). The study was conducted at the Animal Experiment Application and Research Center, Harran University (Şanlıurfa, Türkiye), between September and November 2020. This study was approved by Harran University Animal Experiments Local Ethics Committee with number 2019/002/02.

### 2.4. Experimental Design

The EAT model was established by intraperitoneal inoculation of 3 × 10^5^ EAT cells into each mouse [41]. Different experimental groups received either OLT, balanced salt solution (BSS, formulated by Hanks, Sigma Aldrich, St. Louis, MO, USA), 5-Fluorouracil (5-FU, Sigma Aldrich, St. Louis, MO, USA), or a combination of both, as detailed in Table 1. A dose of 400 mg/kg (body weight) OLT was administered daily by oral gavage, based on individual body weights. This dose, selected in reference to prior in vitro results and LD_50_ data (3000–3475 mg/kg) [42,43], was freshly prepared each day. Commercially available 5-FU was injected intraperitoneally as a single dose at 20 mg/kg body weight [44]. The injection volume for each mouse was adjusted according to its current body weight, as specified in Table 1.

### 2.5. Ascites Fluid Collection and Assessment of Tumor Cell Proliferation

On days 8 and 15 of the experiment, ascites fluid containing tumor cells was collected from the peritoneal cavity of mice anesthetized with ketamine hydrochloride (Ketalar^®^, 80 mg/kg body weight, i.m., Bayer, Leverkusen, Germany) and xylazine hydrochloride (Rompun^®^, 10 mg/kg body weight, i.m., Bayer, Leverkusen, Germany). The total volume of the ascites fluid was measured, and viable EAT cells were quantified using the Trypan blue exclusion assay [45]. Trypan blue was obtained from a commercial source (Gibco, Thermo Fisher Scientific, Waltham, MA, USA). This method allowed for the determination of live EAT cell counts per milliliter, thereby evaluating the effect on tumor cell proliferation.

### 2.6. Tissue Collection

At the end of the experiment, liver, brain, kidney, bladder, and duodenum tissues were collected under sterile conditions for biochemical, molecular, and histopathological analyses. Biochemical assays were performed on liver, kidney, and brain tissues using spectrophotometric methods with commercial kits. For miRNA analysis, liver samples, the body’s primary detoxification center, were placed in RNAlater, flash-frozen in liquid nitrogen, and stored for molecular evaluation. For histopathology, liver, stomach, duodenum, kidney, and bladder tissues were fixed in 10% neutral buffered formalin (Merck, Darmstadt, Germany), embedded in paraffin (Paraplast plus^®^, The Fluka Biochemika, Sigma Aldrich, St. Louis, MO, USA), and stained with hematoxylin and eosin (H&E) (Merck, Darmstadt, Germany) for microscopic examination.

### 2.7. miRNA Expression Analysis

The expression of miR-155-5p in liver samples was measured using reverse transcription quantitative PCR (RT-qPCR). Approximately 25 mg of liver tissue was processed to isolate total RNA, including miRNAs, with QIAzol lysis reagent and the miRNeasy Mini Kit (Qiagen, Germantown, MD, USA). RNA yield and purity were determined using a Nanodrop spectrophotometer (MultiSkan IT, Thermo Fisher Scientific, Waltham, MA, USA). Complementary DNA was synthesized via the miScript II RT Kit, and amplification was carried out using the miScript SYBR Green PCR Kit on a BioRad CFX Connect platform (Bio-Rad Laboratories, Hercules, CA, USA). RNU6B served as the reference gene, and relative expression values were obtained through the 2^−ΔΔCt^ calculation method [46].

### 2.8. Biochemical Experiments

#### 2.8.1. Tissue Sample Preparation

Liver, kidney, and brain tissues were homogenized in 0.1 M potassium phosphate buffer (pH 7.4) using a Teflon homogenizer (Heidolph RZR 2021; Heidolph, Schwabach, Germany). Homogenates were centrifuged at 16,000× *g* for 20 min at 4 °C (Hettich 460 R; Andreas Hettich GmbH & Co. KG, Tuttlingen, Germany), and the resulting supernatants were collected for enzymatic and protein analyses. All assays were performed in triplicate on the same day using a microplate reader (Varioskan Flash 2000; Thermo Fisher Scientific, Waltham, MA, USA) without freezing the samples.

#### 2.8.2. Determination of Enzymatic and Non-Enzymatic Biomarkers

Enzyme activities and total protein concentrations were measured using a microplate reader (Varioskan Flash 2000; Thermo Fisher Scientific, Waltham, MA, USA) immediately after tissue homogenization and centrifugation. Each measurement was performed in triplicate, and absorbance values differing by more than 10% were repeated. Enzyme activities were expressed as specific activity (nmol/min/mg total protein) following total protein quantification. Carboxylesterase (CaE) activity was determined using a modified method of Santhoshkumar & Shivanandappa (1999), with p-nitrophenyl acetate (PNPA) as the substrate [47]. Samples were incubated with Tris buffer (pH 7.4) and PNPA, and absorbance was measured at 405 nm. Glutathione S-transferase (GST) activity was assessed using the method of Habig et al. (1974) with CDNB and reduced glutathione as the substrate and the cofactor, respectively [48]. Absorbance was measured at 344 nm. Alanine aminotransferase (ALT) and aspartate aminotransferase (AST) activities were determined using commercial kits (Biolabo 80,027; 80,025; and 92,111; Biolabo SA, Maizy, France) [49]. Reduced Glutathione (GSH) content was measured based on the method of Moron et al. (1979), with absorbance read at 412 nm [50]. Results were expressed as nmol GSH/mg protein. Total protein concentrations were determined using the Bradford method [51]. Absorbance was measured at 595 nm, and protein content was calculated considering the dilution factor.

### 2.9. Histopathologic Evaluation

Liver, stomach, duodenum, kidney, and bladder tissues from experimental groups were evaluated histologically using light microscopy. Tissue samples were fixed in 10% neutral-buffered formalin, placed into paraffin, and sliced to a thickness of 4 μm. Sections were stained with hematoxylin and eosin (H&E) following a protocol adapted from Cardiff et al. (2014) [52]. After deparaffinization and rehydration through graded alcohols, nuclei were stained with hematoxylin (5 min), followed by eosin counterstaining (1 min). Slides were dehydrated, cleared in acetone and xylene, and mounted with Entellan. Histological examinations were performed under a light microscope, and representative images were recorded.

### 2.10. Statistical Analysis

Statistical evaluations were conducted using GraphPad Prism software (version 10.5.0, GraphPad Software, version 10.5.0, GraphPad Software, San Diego, CA, USA). For datasets following a normal distribution, either one-way ANOVA with Tukey’s multiple comparison procedure or an unpaired Student’s *t*-test was employed. In cases where the data were non-normally distributed, the Kruskal–Wallis test was used, followed by Dunn’s multiple comparison test.

## 3. Results and Discussion

### 3.1. Olive Leaf Tea Reduces Breast Cancer Formation

**Figure 1:**
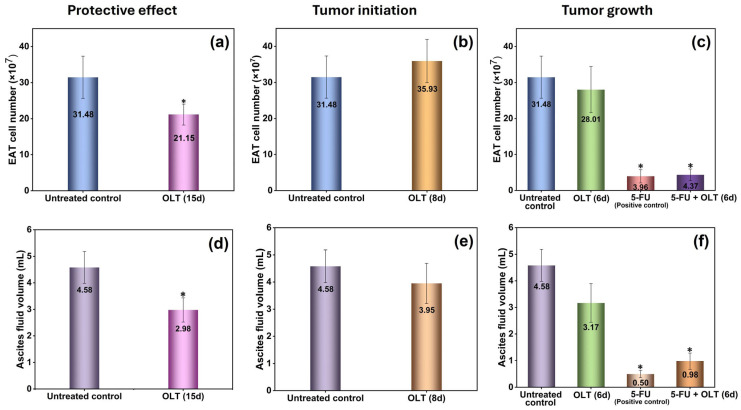
(**a**–**c**) EAT cell numbers and (**d**–**f**) ascites fluid volumes, in mice given 400 mg/kg OLT for 15 days (protective effect), 8 days (tumor initiation), and 6 days (tumor growth), respectively. *: Statistically significant difference compared to untreated control (*p* < 0.05).

The present study demonstrates that oral administration of OLT exerts a protective effect against breast cancer development, as evidenced by its impact on EAT progression in a murine model. To assess the effect of OLT on EAT progression, total cell numbers in the ascites fluid were quantified using trypan blue staining to distinguish viable and non-viable cells. As shown in Figure 1a,d, daily oral administration of 400 mg/kg OLT, starting 7 days prior to tumor inoculation and continuing for 15 days, resulted in a statistically significant reduction in both tumor cell count and ascites fluid volume. The total EAT cell count in this group decreased from 31.48 × 10^7^ in the untreated control to 21.15 × 10^7^ (*p* < 0.05), indicating a significant inhibition of tumor growth (Figure 1a). Simultaneously, the ascites fluid volume was significantly reduced from 4.58 mL to 2.98 mL (*p* < 0.05), further supporting the suppressive effect of OLT on tumor burden (Figure 1d).

These findings align with existing literature on the anticancer properties of olive leaf constituents. Bioactive polyphenols in olive leaves, such as oleuropein, hydroxytyrosol, and verbascoside, have been shown to exert antiproliferative, pro-apoptotic, and anti-angiogenic effects in various cancer models, including breast cancer [12,13,14,15,22,23,24]. Bulotta et al. (2014) reported that oleuropein and hydroxytyrosol significantly reduce the viability of breast cancer cells by modulating oxidative stress and inhibiting oncogenic signaling pathways [12]. Similarly, Elamin et al. (2013) found that oleuropein induces apoptosis in MCF-7 breast cancer cells by activating caspases and modulating the mitochondrial pathway [13]. Moreover, an in vivo study demonstrated that administration of olive leaf extract resulted in a reduction in tumor size and enhanced expression of apoptotic markers in mice bearing breast tumors [53]. These findings from the literature corroborate the antitumor activity observed in the present study. In our model, it has been determined that OLT exerted a protective effect when administered before tumor formation, but had no inhibitory effect on tumor growth when administered on the same day as tumor formation (tumor initiation) or after tumor detection (tumor growth). The group administered OLT for 8 days, starting from the day of tumor inoculation, showed a nonsignificant difference in cell count (35.93 × 10^7^, *p* > 0.05) compared to the control (Figure 1b). As observed on Figure 1c, when OLT administration commenced on the second day post-tumor inoculation and continued for 6 days, the total cell count was 28.01 × 10^7^. It was statistically nonsignificant reduction compared to the control (*p* > 0.05). As a positive control, a single dose of 5-FU, a chemotherapeutic agent commonly used in the treatment of breast cancer, significantly decreased the cell count to 3.96 × 10^7^ (*p* < 0.05). Notably, the combined therapy of 5-FU with OLT resulted in a cell count of 4.37 × 10^7^, demonstrating a statistically significant reduction compared to the untreated control (*p* < 0.05). Additionally, the ascites fluid volume showed a similar trend with cell number (Figure 1e,f). In the groups administered OLT for 8 days, starting from the day of tumor inoculation or commenced on the second day post-tumor inoculation and continued for 6 days, there were statistically nonsignificant changes compared to the control in ascites fluid volume. Groups treated with 5-FU alone or in combination with OLT exhibited markedly lower ascitic fluid volumes, each measuring less than 1 mL, with statistically significant differences from the control group (*p* < 0.05).

### 3.2. Olive Leaf Tea Supports Overall Health

#### 3.2.1. Body Weight

**Figure 2:**
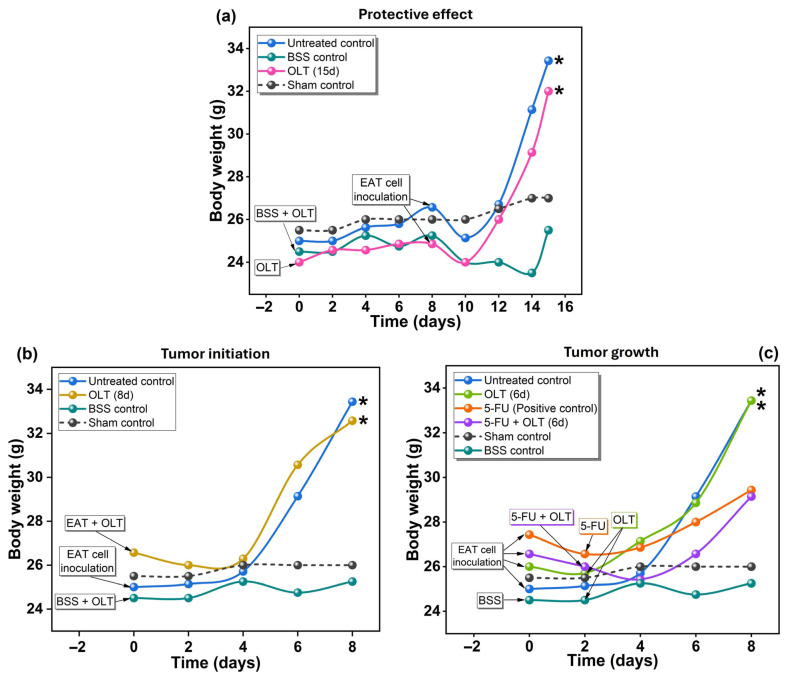
Body weights of animals that were treated with OLT for (**a**) 15, (**b**) 8, and (**c**) 6 days. *: Statistically significant difference compared to sham control (*p* < 0.05).

Body weight changes were monitored over 15 days to assess tumor progression and systemic effects of OLT treatment in mice for different durations (6, 8, or 15 days). As illustrated in Figure 2, the sham and BSS (vehicle) control groups maintained stable weights (25.0–27.0 g). At the same time, EAT-bearing mice showed a significant increase in weight from day 4 due to ascites accumulation and tumor cell proliferation (*p* < 0.05). The rate of body weight gain was calculated and statistically analyzed among the groups. A significant increase in body weight gain (*p* < 0.05) was observed in the EAT-bearing mice compared to the sham control group, primarily due to ascitic fluid accumulation and tumor proliferation. In contrast, OLT-treated groups showed a markedly lower body weight gain rate than the untreated EAT group (*p* < 0.05), indicating that OLT administration effectively limited ascitic fluid formation.

Throughout the experimental period, no abnormalities in food or water intake, grooming behavior, or locomotor activity were observed among any of the groups. Additionally, no signs of systemic toxicity such as lethargy, piloerection, or impaired motor coordination were noted. These findings suggest that the observed body weight gain in the EAT-bearing mice was mainly attributable to ascitic fluid accumulation and tumor cell proliferation, rather than to metabolic or behavioral alterations. These observations align with previous findings in EAT models, in which ascites fluid buildup is a hallmark of rapid tumor progression [54].

In the OLT (15 d) group, where treatment began before tumor inoculation, body weight was significantly higher than in the sham control group (*p* < 0.05). However, there was no statistically significant difference in body weight gain between the OLT (15 d) group and the untreated EAT group (*p* > 0.05). Similarly, the OLT (8 d) and OLT (6 d) groups, where treatment started at or after tumor inoculation, exhibited significantly higher body weights compared to the sham control (*p* < 0.05) but did not differ significantly from the untreated EAT group (*p* > 0.05). These findings suggest that while OLT treatment did not significantly prevent the increase in tumor-related body weight, the trend toward a slower gain in the OLT (15 d) group may still reflect a mild protective or metabolic effect, warranting further investigation. It should be noted that, although olive oil differs from olive leaf extract in its primary constituents, being rich in triglycerides and fatty acids rather than polyphenols, previous studies have reported that diets high in olive oil exhibit protective effects against experimental breast cancer formation in animals [55]. This observation, while not directly comparable in composition, supports the notion that *Olea europaea*-derived products may exert chemopreventive effects through distinct but complementary mechanisms. Boss et al. (2016) and Pessoa et al. (2024) reported that pre-treatment of olive leaf extracts may delay tumor development by regulating cellular pathways involved in inflammation and oxidative stress [9,56]. In the present study, the reduction in tumor-associated weight gain in the OLT (15 d) group may result from olive leaf extract’s known ability to inhibit tumor cell viability, reduce angiogenesis, and suppress pro-inflammatory cytokines, all of which contribute to ascites formation [57].

#### 3.2.2. Liver and Gastrointestinal System

**Figure 3:**
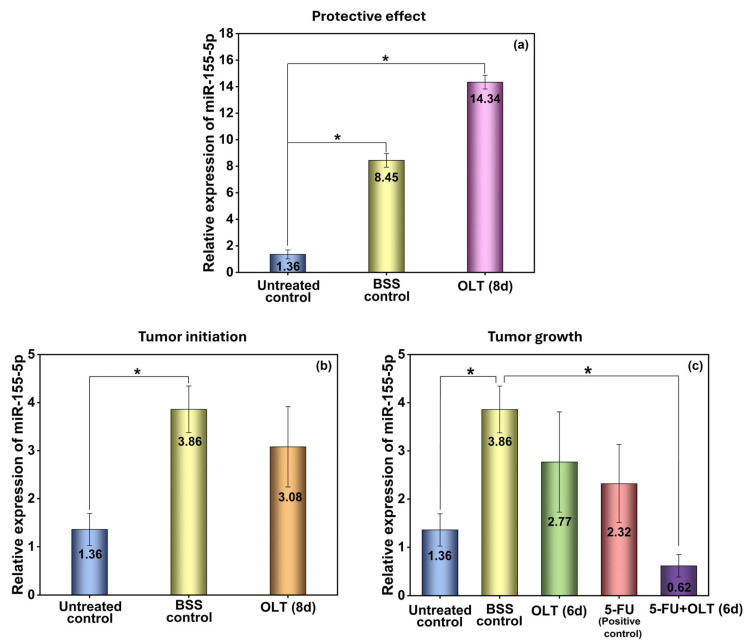
Relative miR-155-5p expression levels of the liver obtained from animals that were treated with OLT for (**a**) 15, (**b**) 8, and (**c**) 6 days. *: Statistically significant difference between groups (*p* < 0.05).

This study demonstrates that OLT supplementation has a multifaceted protective effect on liver function and oxidative status in mice with EAT, particularly when administered in a prophylactic manner. One of the most striking findings was the strong upregulation of miR-155-5p expression in the livers of animals treated with OLT for 15 days, starting 7 days before tumor induction; there was a 14.34-fold increase compared to the untreated control group (*p* < 0.05) (Figure 3). This marked elevation suggests that early OLT intervention can activate molecular pathways associated with tumor suppression. Moreover, in the hepatic environment, miR-155 has been reported to reduce inflammation and modulate lipid metabolism, contributing to tissue homeostasis [33]. Interestingly, while 5-FU alone induced a slight increase in miR-155-5p expression, possibly due to an inflammatory response, the addition of OLT attenuated this elevation, though the decrease was not statistically significant. This trend suggests that OLT may fine-tune miRNA-mediated inflammatory pathways during chemotherapy. A previous study has shown that excessive miR-155 expression in response to chemotherapeutic stress can disrupt immune regulation and exacerbate tissue injury [58].

miR-155 is a well-documented pleiotropic regulator with oncogenic and tumor suppressive functions depending on the tissue context, inflammatory milieu, and tumor stage [28]. It has been observed in multiple cancer types that elevated miR-155 expression promoted tumorigenesis by enhancing cellular proliferation, migration, and immune evasion mechanisms, showing its oncogenic roles [59,60,61]. Contrary to this, miR-155 exhibited tumor-suppressive effects by modulating inflammatory responses, reducing oxidative stress, and facilitating apoptosis in specific tissues [60]. Recent studies have clearly shown a dual role: miR-155 overexpression is associated with aggressive phenotypes in hematological malignancies, while its increase is also linked to better prognosis and lower tumor burden in liver and colorectal cancers due to its regulation of inflammatory signaling pathways [59].

In our model, the dual modulation of miR-155 by OLT, upregulation in the OLT-only group and downregulation in the 5-FU + OLT group, should be interpreted within its immunomodulatory and chemoresistance-related roles rather than as a uniform oncogenic response. Elevated miR-155 has been shown to enhance antitumor immunity by promoting CD8^+^ T-cell cytotoxicity and IFN-γ production [58], whereas excessive miR-155 can foster chemoresistance and inflammatory toxicity [62]. Accordingly, the increase in miR-155 with OLT alone may signify immune activation consistent with OLT’s polyphenolic, immunoregulatory properties, while its reduction under combined 5-FU + OLT treatment likely reflects a beneficial normalization of stress-responsive pathways.

**Figure 4:**
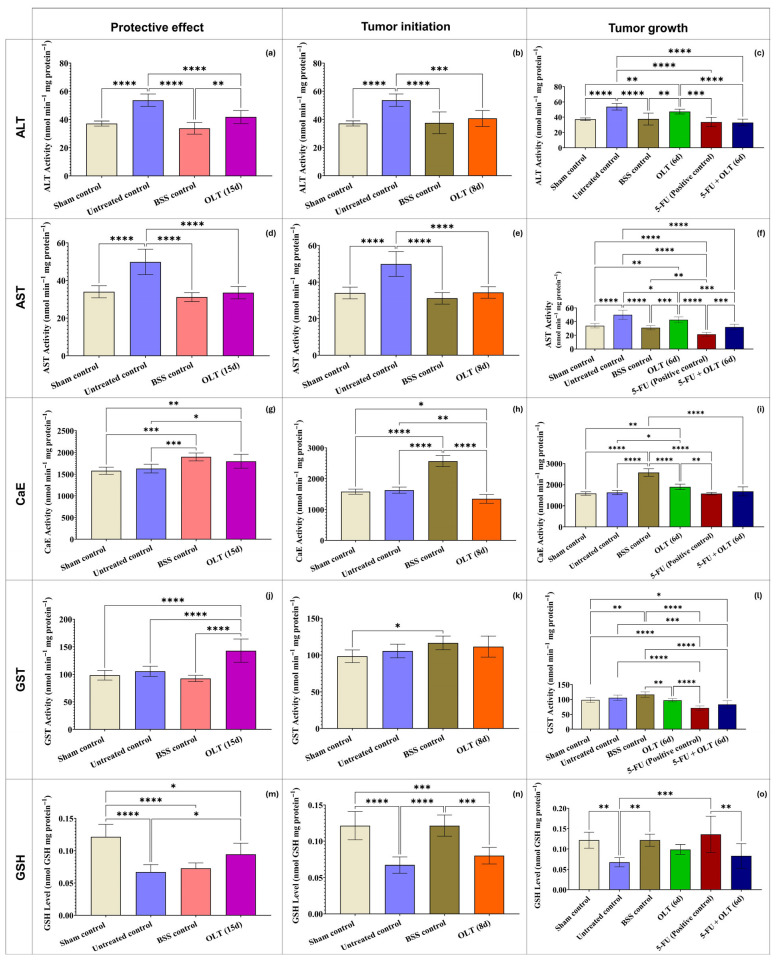
In liver tissues of mice treated with 400 mg/kg OLT for 15, 8, or 6 days (**a**–**c**) ALT; (**d**–**f**) AST; (**g**–**i**) CaE; (**j**–**l**) GST, (**m**–**o**) GSH levels. Statistically significant differences between groups indicate with asterisks (*: *p* < 0.05; **: *p* < 0.01; ***: *p* < 0.001; ****: *p* < 0.0001).

In case of hepatocellular damage caused by tumor-related oxidative stress, free radicals initiate lipid peroxidation, which causes cell membrane deterioration in tissues. As a result, enzymes such as alanine aminotransferase (ALT), aspartate aminotransferase (AST), and alkaline phosphatase (ALP) are secreted into the interstitial fluids [63]. In this study, the elevation in miR-155 expression coincided with significant reductions in ALT and AST levels. Although these findings suggest a possible association between miR-155 modulation and hepatic protection, the present data indicate a correlative rather than a causal relationship, as no functional assays were performed to demonstrate that miR-155 directly drives changes in liver enzyme activity. While ALT and AST were significantly elevated in the untreated tumor-bearing group, OLT administration reduced ALT and AST activities in a duration-dependent manner (Figure 4a–f). For example, ALT decreased from 53.68 nmol/min/mg protein in the untreated control to 41.75 nmol/min/mg protein in the OLT (15 d) group (*p* < 0.001), while AST levels declined from 49.85 nmol/min/mg protein to 33.50 nmol/min/mg protein (*p* < 0.0001), approaching the values observed in the sham control group (33.90 nmol/min/mg). These findings suggest that OLT effectively protects against hepatocellular injury. Possibly, OLT might achieve it through the stabilization of hepatocyte membranes and attenuation of lipid peroxidation, which is a significant cause of membrane leakage [63,64].

Carboxylesterase (CaE) activity, a key enzyme involved in hepatic xenobiotic detoxification, showed an overall increase in OLT-treated groups compared to the untreated control (1630.27 nmol/min/mg protein) and the sham control (1580.31 nmol/min/mg protein). The highest activity was observed in the OLT (6 d) group (1898.14 nmol/min/mg protein, *p* < 0.05, vs. untreated control), followed by OLT (15 d) (1798.41 nmol/min/mg protein, *p* < 0.05, vs. untreated control). These results revealed that the OLT administration enhances hepatic detoxification capacity in a time-dependent manner (Figure 4g–i). This unexpected elevation may suggest the involvement of apoptosis-related pathways. This phenomenon has been previously reported, with studies demonstrating a concurrent increase in both apoptosis and CaE activity following exposure to gold nanorods and ethanol [65,66]. These alterations in CaE activity suggest an adaptive hepatic response to oxidative and metabolic stress. The CaE functions in close coordination with glutathione-dependent antioxidant mechanisms. Therefore, to understand how OLT affects other components of the detoxification system, glutathione (GSH) levels and glutathione S-transferase (GST) activity were evaluated. The antioxidant response observed in the OLT groups was further supported by elevated GSH levels and increased activity of GST, both key components of the cellular redox defense system. GST activity significantly increased in the OLT (15 d) group (142.82 nmol/min/mg protein, *p* < 0.0001, vs. all control groups). This was reflecting enhanced detoxification capacity and improved hepatic redox homeostasis (Figure 4j–l). In parallel, glutathione (GSH) levels, serving as a primary non-enzymatic antioxidant, were notably depleted in the untreated control group (0.067 nmol/mg protein), indicating oxidative imbalance. However, OLT administration restored GSH concentrations in the OLT (15 d) group (0.094 nmol/mg protein, *p* < 0.05, vs. the untreated control group). Together, these findings demonstrate that prolonged OLT supplementation effectively supports the hepatic antioxidant defense system by modulating both enzymatic (GST) and non-enzymatic (GSH) components (Figure 4m–o). This demonstrated that OLT not only prevents oxidative damage but also restores antioxidant capacity. In a study, it was reported that olive leaf extract reversed oxidative damage in rats exposed to lead acetate or ethanol by restoring antioxidant enzymes and lipid peroxidation levels, and improved damaged testicular tissues [67]. It is postulated that the induction of GST and CaE enzymes plays a crucial role in mitigating oxidative stress and counteracting toxicity. The normalization of CaE and GST activities during the recovery phase may be interpreted as a biomarker of recovery, reflecting the attenuation of carcinogen-induced stress [68].

Nutritional factors are crucial in the development, advancement, and prevention of cancer. Olive leaves contain several bioactive compounds such as oleuropein, hydroxytyrosol, and oleic acid that play essential roles in enhancing the body’s antioxidant defense mechanisms and maintaining cellular membrane integrity [69,70]. These phytochemicals, particularly polyphenols and monounsaturated fatty acids (MUFAs, e.g., oleic acid), are known to reduce oxidative stress by inhibiting DNA oxidation and promoting the activity of endogenous antioxidants like glutathione (GSH) [64,71]. Based on our findings, it is probable that these compounds likely contributed to the observed elevation in GSH levels, the normalization of detoxifying enzyme activities, and the reduction in tumor burden, thus suggesting their potential role in modulating carcinogenesis and supporting hepatic function.

**Figure 5:**
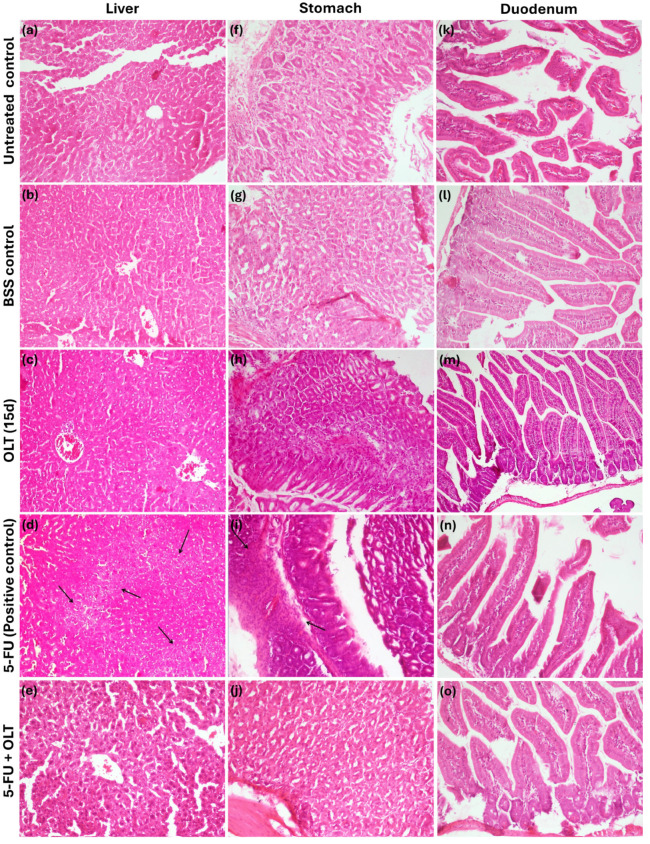
Histopathological evaluation results of liver (**a**–**e**), stomach (**f**–**j**), and duodenum (**k**–**o**). Arrows in tissue sections obtained from 5-FU-treated animals indicate: cytoplasmic vacuolization and nuclear pyknosis in the liver; and focal lymphocyte infiltration in the stomach. (Magnification: 200×).

In addition to biochemical parameters, histopathological findings provided morphological confirmation of OLT’s hepatoprotective effect (Figure 5a–e). While no pathological alterations were noted in the livers of mice treated solely with OLT (400 mg/kg/day), the 5-FU (chemotherapy agent) group exhibited mild hepatocellular degeneration, such as cytoplasmic vacuolization and nuclear pyknosis, which are hallmarks of drug-induced hepatotoxicity [72,73]. Additionally, no pathological findings were observed in the stomach (Figure 5f–j) and duodenum (Figure 5k–o) tissues of animals administered OLT orally. However, in animals administered 5-FU alone, mild focal lymphocyte infiltration was observed, consistent with the side effects of chemotherapeutic agents [74]. Especially, animals receiving both 5-FU and OLT exhibited mitigated histopathological alterations. This suggested a protective synergy. This recovery also showed the normalization of ALT and AST levels, indicating a systemic protective effect against 5-FU-induced hepatic damage.

In summary, the data collectively indicate that OLT, particularly when administered before tumor initiation, offers substantial protective effects against hepatic oxidative damage and tumor progression. These effects are mediated through the upregulation of miR-155, restoration of antioxidant capacity (GSH, GST, CaE), and normalization of liver enzyme profiles. Moreover, OLT demonstrates potential as an adjunct therapy in chemotherapeutic settings, ameliorating drug-induced hepatotoxicity and possibly modulating miRNA-driven immune responses.

#### 3.2.3. Excretory System: Kidneys and Bladder

**Figure 6:**
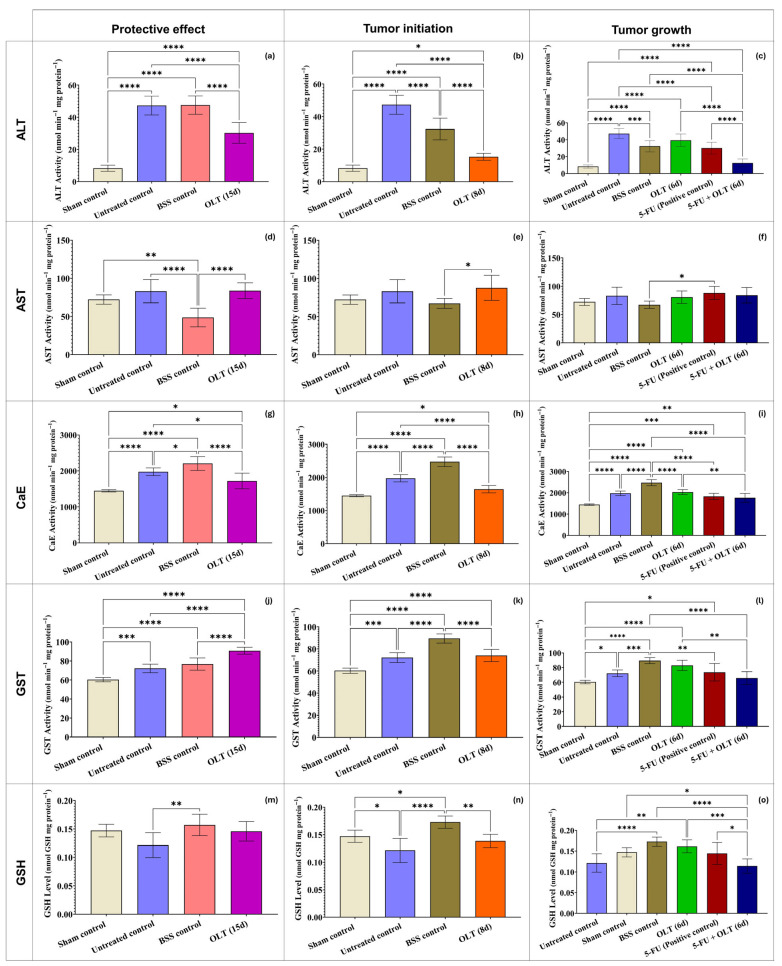
In kidney tissues of mice treated with 400 mg/kg OLT for 15, 8, or 6 days (**a**–**c**) ALT; (**d**–**f**) AST; (**g**–**i**) CaE; (**j**–**l**) GST, (**m**–**o**) GSH levels. Statistically significant differences between groups indicate with asterisks (*: *p* < 0.05; **: *p* < 0.01; ***: *p* < 0.001; ****: *p* < 0.0001).

In tumor-bearing mice, a significant increase in renal ALT activity (47.27 nmol/min/mg protein) was observed compared to sham controls (8.29 nmol/min/mg protein), showing pronounced renal stress associated with tumor progression (*p* < 0.0001). ALT levels reduced in the OLT-treated groups [OLT (8 d): 15.33 nmol/min/mg protein, OLT (15 d): 30.28 nmol/min/mg protein, *p* < 0.0001, vs. untreated control]. The combined 5-FU + OLT (6 d) treatment produced an even greater reduction (12.50 nmol/min/mg protein, *p* < 0.0001, vs. untreated control), suggesting a synergistic effect between OLT and 5-FU chemotherapy (Figure 6a–c). In contrast, AST levels remained relatively consistent across groups, ranging from 72.29 to 88.19 nmol/min/mg protein, without statistically significant changes (Figure 6d–f). This stability likely reflects the mitochondrial localization and slower turnover rate of AST under oxidative stress conditions [75]. These findings align with previous reports highlighting the antioxidant and anti-inflammatory potential of olive-derived polyphenols such as oleuropein and hydroxytyrosol in protecting renal tissues against chemical and metabolic injury [76,77]. CaE activity was elevated in all groups compared to the sham control (1447.38 nmol/min/mg protein). The highest CaE enzyme activity was detected in the BSS control group. This may be due to a process involving apoptosis-related pathways, as described in Section 3.2.2 [65,66]. When comparisons were made between patient groups, it was found that OLT treatment normalized CaE levels, particularly in the 8-day (1645.42 nmol/min/mg protein) and 15-day (1720.21 nmol/min/mg protein) groups compared to the untreated control (tumor) group (1975.30 nmol/min/mg protein) (*p* < 0.05). (Figure 6g–i). These findings suggest a partial renoprotective role for OLT, normalization of detoxification pathways. GST activity increased in both the untreated control (72.21 nmol/min/mg protein, *p* < 0.001, vs. sham control) and the BSS groups (8 d: 89.50 nmol/min/mg protein, 15 d: 76.74 nmol/min/mg protein, *p* < 0.0001, vs. sham control), displaying a stress response. Interestingly, long-term OLT treatment (15 d) led to the highest GST activity (90.83 nmol/min/mg protein, *p* < 0.0001, vs. all control groups), while 5-FU + OLT co-treatment reduced it (65.80 nmol/min/mg protein), possibly reflecting a balanced redox state (Figure 6j–l). Similarly, renal GSH levels decreased from 0.147 nmol/mg protein in the sham control to 0.121 nmol/mg protein in the untreated control group (*p* < 0.05), indicating oxidative imbalance. OLT administration markedly improved GSH concentrations, with the highest level observed in the OLT (6 d) group (0.161 nmol/mg protein, *p* < 0.01 vs. untreated control). In a similar trend, GSH levels were detected in the OLT (8 d) and OLT (15 d) groups as 0.139 nmol/mg protein and 0.146 nmol/mg protein, respectively (*p* > 0.05) (Figure 6m–o). These findings are consistent with previous reports demonstrating that olive leaf polyphenols enhance GSH biosynthesis and recycling, thereby strengthening the antioxidant defense system [78].

**Figure 7:**
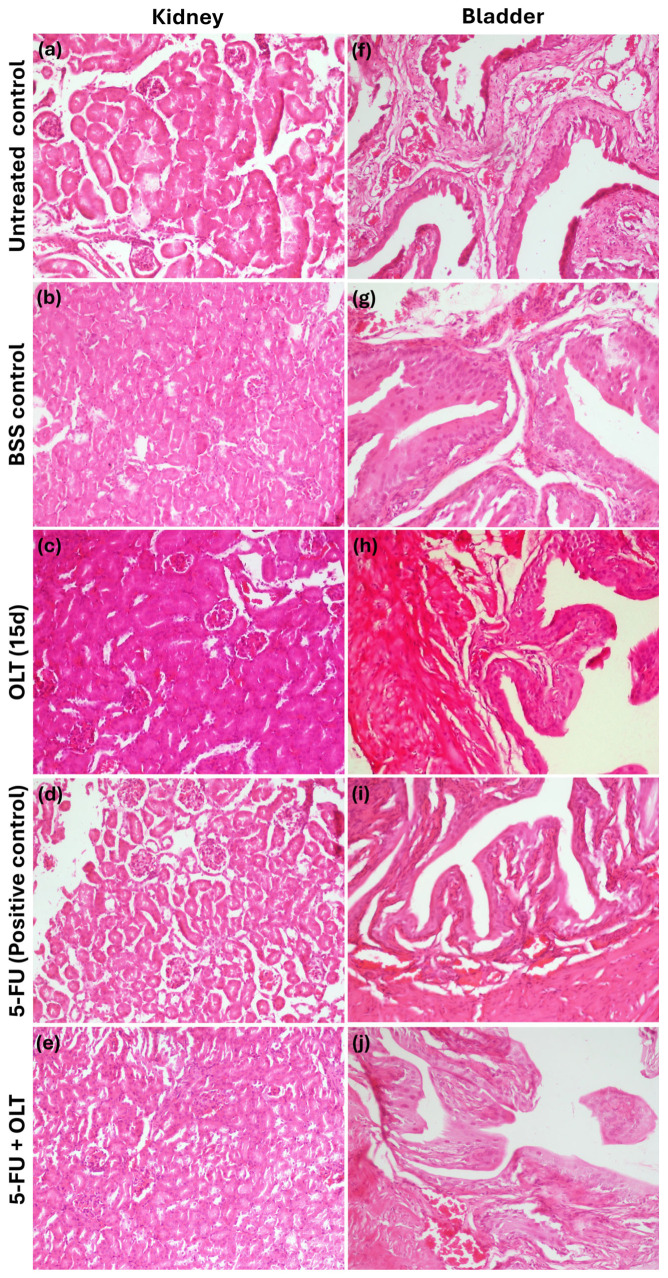
Histological section images from kidney (**a**–**e**) and bladder (**f**–**j**) tissues (Magnification: 200×).

As displayed in Figure 7, histological analysis of kidney (a–e) and bladder (f–j) tissues revealed no pathological changes in any group, including those treated with OLT, 5-FU, or their combination. This absence of structural damage confirms the biocompatibility and renal safety of OLT, even under chemotherapeutic stress.

Taken together, the findings demonstrate that OLT exerts beneficial effects on renal oxidative balance and detoxification pathways under tumor and treatment-related stress. It significantly lowers ALT levels, restores CaE and GST activities, enhances antioxidant GSH capacity, and preserves renal and bladder tissue integrity. The combination of OLT with 5-FU further potentiates these protective effects without inducing nephrotoxicity, highlighting its promise as a safe and effective supportive therapy during cancer treatment.

#### 3.2.4. Brain

In this part of the study, biochemical analyses were performed using whole-brain homogenates to evaluate alterations in enzyme activities related to oxidative stress and metabolic function.

**Figure 8:**
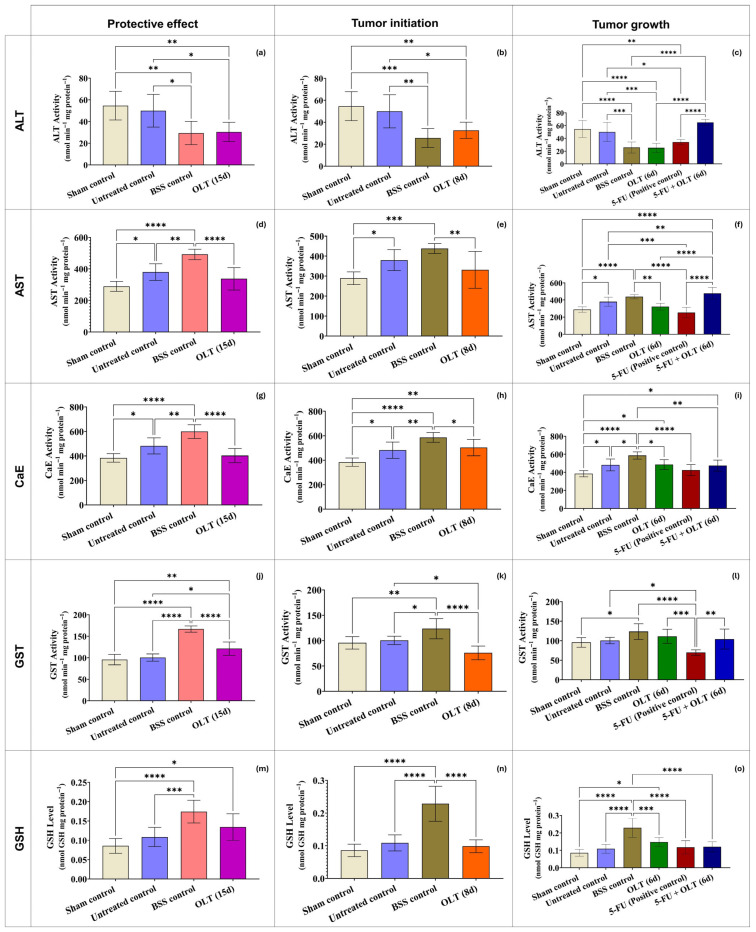
In brain tissues of mice treated with 400 mg/kg OLT for 15, 8, or 6 days (**a**–**c**) ALT; (**d**–**f**) AST; (**g**–**i**) CaE; (**j**–**l**) GST, (**m**–**o**) GSH levels. Statistically significant differences between groups indicate with asterisks (*: *p* < 0.05; **: *p* < 0.01; ***: *p* < 0.001; ****: *p* < 0.0001).

AST levels in brain tissue were significantly elevated in the untreated control group (380.00 nmol/min/mg protein) compared to the sham control (289.43 nmol/min/mg protein, *p* < 0.05), whereas ALT levels were lower in the untreated control group (Figure 8a–f). These findings suggest that mitochondrial stress or neuroinflammation may be a response to tumor burden because AST is more abundant in mitochondria-rich tissues and can reflect cellular damage under systemic oxidative stress [75]. Interestingly, while 5-FU treatment alone reduced AST levels (252.68 nmol/min/mg protein, *p* < 0.001, vs. untreated control), co-administration with OLT led to a significant increase in AST (477.64 nmol/min/mg protein, *p* < 0.001 vs. both sham control and untreated control). This finding suggests a possible exacerbation of the oxidative or metabolic response due to the interaction between OLT polyphenols and chemotherapeutic agents [79]. Conversely, OLT alone treatment (6, 8, and 15 d) resulted in reductions in ALT levels (*p* < 0.05, vs. the sham control and the untreated control groups). This result might indicate a protective effect on neuronal metabolic integrity. Various olive polyphenols have been reported to protect nervous tissue by regulating oxidative enzymes and preserving mitochondrial function [80]. Additionally, it has been shown that oleuropein enhances mitochondrial function by activating the Nrf2 pathway, reducing ROS production, and preserving mitochondrial DNA integrity [81].

In terms of detoxification enzymes, brain carboxylesterase (CaE) activity was elevated in all groups relative to the sham control (384.11 nmol/min/mg protein), with the highest activity observed in the BSS (15 d) control group (599.00 nmol/min/mg protein, *p* < 0.0001) (Figure 8g–i). Interestingly, OLT treatment in tumor-bearing groups slightly reduced this elevation. In the literature, it has been recorded that esterases can up- or down-regulate in stress as a systemic xenobiotic response [82,83].

As shown in Figure 8j–l, GST activity was elevated in all OLT-treated groups, with the OLT (15 d) group (121.15 nmol/min/mg protein) exhibiting a significant increase (*p* < 0.01) compared to the sham control (95.63 nmol/min/mg protein). Although 5-FU reduced GST activity (69.58 nmol/min/mg protein), its co-administration with OLT restored levels to 104.09 nmol/min/mg protein. This indicates an alleviating effect of OLT against chemotherapy-induced oxidative stress. Similar findings were reported by Dzah et al. (2024), who demonstrated that polyphenolic compounds can restore GST activity under drug-induced stress conditions [84]. Moreover, previous studies have shown that olive leaf extract counteracts chemotherapy-associated suppression of antioxidant enzymes [85]. Regarding GSH, no significant differences were observed between the sham (0.086 nmol GSH/mg protein) and untreated control (0.109 nmol GSH/mg protein) groups. However, GSH concentrations increased in OLT-treated groups, reaching 0.147 nmol GSH/mg protein in the OLT (6 d) group (*p* < 0.05) (Figure 8m–o). As a key non-enzymatic antioxidant, GSH plays a crucial role in maintaining redox homeostasis within the central nervous system [86]; thus, its elevation underscores the neuroprotective potential of olive leaf polyphenols [87]. Interestingly, the absence of significant GSH alterations between tumor-bearing and sham animals suggests the activation of compensatory antioxidant mechanisms in the brain under moderate systemic stress.

Taken together, these results indicate that OLT exerts subtle but beneficial effects on brain oxidative metabolism, particularly by modulating GST and GSH levels and maintaining enzyme activity profiles under tumor or chemotherapy stress. While the 5-FU + OLT combination showed unexpected elevations in aminotransferase levels, likely reflecting complex drug-nutrient interactions, OLT alone demonstrated a favorable profile. This supports the growing evidence that olive-derived polyphenols may serve as neuroprotective agents through antioxidant and anti-inflammatory mechanisms [87,88].

### 3.3. Antioxidant and Phenolic Characterization of Olive Leaf Tea

To further elucidate the in vivo biological effects of OLT, its antioxidant potential was evaluated through in vitro assays, which revealed a strong antioxidant capacity. Specifically, the Ferric Reducing Antioxidant Power (FRAP) was measured at 81.23 ± 3.50 µmol Fe^2+^/g, and the DPPH radical scavenging activity was recorded as 197.88 ± 4.83 µmol TE/g. These findings reveal a significant potential for neutralizing free radicals. These results also agree well with previous studies reporting the antioxidant properties of olive leaf infusions. For instance, Ghomari et al. (2019) recorded FRAP values ranging from 60 to 140 µmol Fe(II)/L and DPPH activities from 150 to 220 µg/mL Trolox equivalent, depending on the cultivar and extraction method [89]. In our study, the Total Phenolic Content (TPC) was found to be 25.74 ± 1.63 mg GAE/g dry sample. This finding is consistent with previous reports on aqueous olive-leaf infusions, which ranged between 10.5 and 72.4 mg GAE/g dry plant matter depending on cultivar, drying process, and extraction temperature [89,90,91]. Debib and Boukhatem (2017) reported that TPC was found to be 10.5 mg GAE/g dry matter in the aqueous extract of Tunisian *Chemlali* variety olive leaves [90]. However, Ghomari found varying TPC ratios in different solvents and temperatures, and recorded this value as 45.40 mg GAE/g dry matter for the distilled water extract [89]. Borghini et al. (2024) reported TPC values of 35.7–72.4 g GAE/kg in aqueous extracts of *Olea europaea* leaves [91].

**Figure 9:**
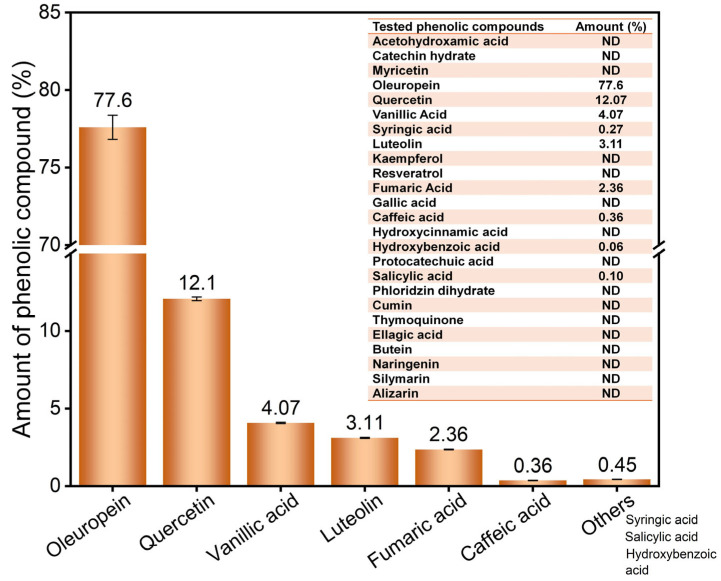
The amount (%) of tested phenolic compounds of olive leaves. ND: Not defined.

LC-MS/MS analysis revealed that oleuropein was the dominant phenolic compound of OLT among the tested phenolic components, comprising 77.60% of the total phenolic content (787.88 mg/kg), followed by quercetin (12.07%), luteolin (3.11%), vanillic acid (4.07%), and others, including fumaric, caffeic, and syringic acids (Figure 9). This phenolic profile is consistent with previous reports that identify oleuropein as the principal bioactive component responsible for the antioxidative, anti-inflammatory, and anticancer effects of olive leaves [91,92]. The predominance of oleuropein provides mechanistic support for the antitumor and hepatoprotective effects observed in this study. It has been reported that oleuropein inhibits cancer cell proliferation, promotes apoptosis, and modulates oxidative stress [13,25]. Additionally, polyphenols such as quercetin and luteolin have been demonstrated to synergistically enhance these biological effects by scavenging free radicals and upregulating phase II detoxification enzymes [93]. Zhang et al. (2024) showed that quercetin has been targeted at Nrf2 and demonstrates robust antioxidant and anti-inflammatory capabilities [94]. Luteolin is a flavone that modulates oxidative stress via the Nrf2 pathway [95,96], further supporting the functional potential of the OLT in vivo.

These biochemical findings align with the in vivo observations of reduced tumor burden, improved redox balance, and protection of liver and kidney function in OLT-treated mice. The potent antioxidant characteristics of the tea likely contributed to the upregulation of hepatic miR-155-5p, normalization of ALT and AST levels, and restoration of GSH and GST activity. Thus, the phenolic profile of OLT not only reinforces its nutraceutical promise but also provides a mechanistic basis for the chemopreventive and systemic protective effects observed in the murine EAT model.

## 4. Conclusions

This study demonstrated that the timing of OLT administration plays a decisive role in its chemopreventive and systemic protective efficacy in the EAT model. Prophylactic OLT treatment, initiated before tumor induction, effectively suppressed tumor progression, maintained systemic homeostasis, and caused no detectable toxicity. In contrast, treatments initiated after tumor formation produced limited effects, highlighting the critical importance of early intervention.

Pre-treatment with OLT, initiated prior to tumor inoculation, significantly reduced viable EAT cell counts and ascites volume, accompanied by hepatic upregulation of miR-155-5p, normalization of liver enzyme activities, and restoration of antioxidant capacity through enhanced GSH and GST levels. Similar protective responses were observed in renal and brain tissues, consistent with OLT’s broad systemic activity and safety profile. Moreover, co-administration of OLT with 5-fluorouracil preserved the drug’s therapeutic efficacy while attenuating hepatotoxicity, suggesting its potential utility in combination chemotherapy. LC-MS/MS analysis identified oleuropein, followed by quercetin and luteolin, as the potential phenolic constituents underlying these biological effects. When administered prophylactically, OLT appears to pre-activate antioxidant and anti-inflammatory pathways via its high polyphenol content, thereby enhancing redox regulation and modulating key microRNAs such as miR-155-5p. This preconditioning strengthens systemic defenses and reduces susceptibility to tumor initiation, whereas once the tumor is established, these regulatory mechanisms become less responsive to OLT intervention.

In conclusion, early OLT supplementation enhances systemic antioxidant capacity and immune regulation, contributing to tumor prevention and organ protection. Given its natural origin, safety, and bioactivity, OLT represents a promising nutraceutical candidate for preventive and adjunctive cancer therapy, warranting further clinical and mechanistic investigations to substantiate these preclinical findings.

## Figures and Tables

**Table 1:** The groups used for the experiments.

Groups		Regime	Number of Mice
Sham control		No operation	4
Untreated control (EAT)		3 × 10^5^ EAT cells, intraperitoneal (i.p.) injection on day 0	7
BSS (healthy) controls	BSS (8 d)	No tumor + BSS, i.p. injection on day 0 + OLT (400 mg/kg/day, orally) on day 0–7 (for 8 days)	4
	BSS (15 d)	No tumor + BSS i.p. injection on day 0 + OLT (400 mg/kg/day, orally) on day 0–14 (for 15 days)	4
Protective effect	OLT (15 d)	OLT (400 mg/kg/day, orally) on day 0–14 (for 15 days) + 3 × 10^5^ EAT cells, i.p. injection on day 7	7
Tumor initiation	OLT (8 d)	3 × 10^5^ EAT cells, i.p. injection on day 0 + OLT (400 mg/kg/day, orally) on day 0–7 (for 8 days)	7
Tumor growth	OLT (6 d)	3 × 10^5^ EAT cells, i.p. injection on day 0 + OLT (400 mg/kg/day, orally) on day 2–7 (for 6 days)	7
	5-FU (Positive control)	3 × 10^5^ EAT cells, i.p. injection on day 0 + 5-FU, i.p. injection on day 2 (single dose)	7
	5-FU + OLT (6 d)	3 × 10^5^ EAT cells, i.p. injection on day 0 + 5-FU, i.p. injection on day 2 (single dose) + OLT (400 mg/kg/day, orally) on day 2–7 (for 6 days)	7

## Data Availability

The original contributions presented in this study are included in the article. Further inquiries can be directed to the corresponding author.

## Abbreviations

The following abbreviations are used in this manuscript:

EAT	Ehrlich Ascites Tumor
OLT	Olive Leaf Tea
5-FU	5-Fluorouracil
BSS	Balanced Salt Solution
ALT	Alanine aminotransferase
AST	Aspartate aminotransferase
CaE	Carboxylesterase
GST	Glutathione S-transferase
GSH	Glutathione
